# LeARN: a platform for detecting, clustering and annotating non-coding RNAs

**DOI:** 10.1186/1471-2105-9-21

**Published:** 2008-01-14

**Authors:** Céline Noirot, Christine Gaspin, Thomas Schiex, Jérôme Gouzy

**Affiliations:** 1 Laboratoire Interactions Plantes Micro-organismes UMR441/2594, INRA/CNRS, F-31320 Castanet Tolosan, France; 2 Unité de Biométrie et d'Intelligence Artificielle UR875, INRA, F-31320 Castanet Tolosan, France

## Abstract

**Background:**

In the last decade, sequencing projects have led to the development of a number of annotation systems dedicated to the structural and functional annotation of protein-coding genes. These annotation systems manage the annotation of the non-protein coding genes (ncRNAs) in a very crude way, allowing neither the edition of the secondary structures nor the clustering of ncRNA genes into families which are crucial for appropriate annotation of these molecules.

**Results:**

LeARN is a flexible software package which handles the complete process of ncRNA annotation by integrating the layers of automatic detection and human curation.

**Conclusion:**

This software provides the infrastructure to deal properly with ncRNAs in the framework of any annotation project. It fills the gap between existing prediction software, that detect independent ncRNA occurrences, and public ncRNA repositories, that do not offer the flexibility and interactivity required for annotation projects. The software is freely available from the download section of the website

## Background

Our knowledge of small non-protein-coding RNAs (ncRNAs) has considerably evolved during the last decade. In 2002, *Science *magazine selected the discovery of small RNA with a regulatory function as a scientific breakthrough of the year [1]. Since, it has been discovered that various forms of ncRNA molecules play an important function in regulating gene expression. First examples include small temporal RNA, or microRNA, that regulates development in *C. elegans *[2]. It is now confirmed that genomes in all kingdoms encode for ncRNA playing regulatory roles [3-5]. Because it is believed that only a small fraction, corresponding to the tip of the iceberg, has been discovered, different approaches including experimental and computational ones have recently been developed in order to identify more ncRNAs.

Detecting novel ncRNAs by experimental RNomics is not an easy task [6]. This has led both to the proposition of alternative computational methods that aim to detect and analyze ncRNAs in genomic sequences and, simultaneously, to an increasing development of generalist and specific RNA databases. These tools cover a broad spectrum of the needs in the RNA field [7]. Currently, Rfam [8] can be considered as the most comprehensive repository of validated ncRNA families and the Infernal[9]/Rfam pair is used in the framework of the majority of genome annotation projects. In addition to the Rfam standard, others databases are available; focusing on one class of molecules (The microRNA Registry [10]), on one genome (ASRP [11]), or aiming at providing an exhaustive catalogue of ncRNAs (NONCODE [12]).

Computational methods for ncRNA prediction can be classified into four approaches; bias composition analysis, minimization of free energy, searching for homologous RNA in the context of conserved family-specific characteristics, and sequence-based homology searches. The first approach analyzes the intrinsic features of the genomic sequence in order to detect ncRNA candidates. These *ab-initio *methods rely on an existing bias in base composition between ncRNA and the rest of the genome in order to provide a segmentation of the genomic sequence into ncRNA regions and others [13,14]. They are often used in combination with available transcription signals detection, structure prediction and comparative approaches. The second approach makes use of RNA folding algorithms in order to select those regions of best free energy. This approach has been very successful in the context of finding miRNA [15]. Candidates are filtered out on the basis of thermodynamic stability estimation [16]. A third approach relies on existing ncRNA families which can be described by their common sequence and structural elements. This approach uses validated sequence/structure alignments in order to identify the key ribonucleotides involved in the molecule structure. Alignments are then processed and modelled in several ways, they can be used to build profiles as implemented in ERPIN [17], or covariance models that are implemented in the Infernal package which is the engine behind the Rfam RNA database. This approach also includes programs dedicated to one type of ncRNA like the widely used tRNAScan-SE [18]. More flexible programs are included in this class, providing users with a programming language allowing the description of any ncRNA structure (RNAMOT [19]; Milpat [20]). These programs require significant expertise of a family. The last approach makes use of the increasing number of genomic sequences which provide a rich dataset for computational comparative sequence analysis. Recent developments such as QRNA [21], RNAz [22], MSARI [23], ddbRNA [24] have highlighted a high number of known and new ncRNA families.

Complementary to the development of detection strategies, many user interfaces have been developed to modify and annotate RNA sequences and structures (ESSA [25], S2S [26], 4SALE [27]). In addition to these binary resources, the RNAML [28] syntax was defined in 2002. The aim of this consensus syntax is to transfer information among the RNA community and to ensure a basic interoperability between programs.

After a decade of genome annotation, many powerful protein annotation platforms have been developed (MANATEE [29], GenDB [30], AGMIAL [31]). In the framework of annotation projects, these platforms fill the gap between existing gene prediction software and public protein databases. However, they deal very poorly with ncRNA genes, relying on limited detection software whose output is displayed in a very crude way.

Moreover, numerous RNAs identified by RNomics or computational screens lack precise known sequence and structural motifs making them difficult to classify into families.

Thus, there is now a clear need for ncRNA gene annotation platforms. Such a platform should provide an interactive environment for the analysis and annotation of ncRNA genes with regard to their sequence and structural conservation within gene families. Also, it should be able to rely on a flexible set of detection methods and provide services such as incremental updates and redundancy removal (which are absolutely required in the context of ongoing sequencing projects).

The aim of LeARN, the annotation platform presented here, is to manage the complete process of annotation of ncRNA genes. It can incrementally integrate the results of arbitrary detection programs and provides life scientists with user friendly interfaces allowing both structural and functional annotations. In order to facilitate later exploitation of annotations, LeARN relies on existing standards such as the Rfam database and the RNAML data exchange format, thus providing full interoperability with existing databases and software.

## Implementation

### Architecture

**Figure 1:**
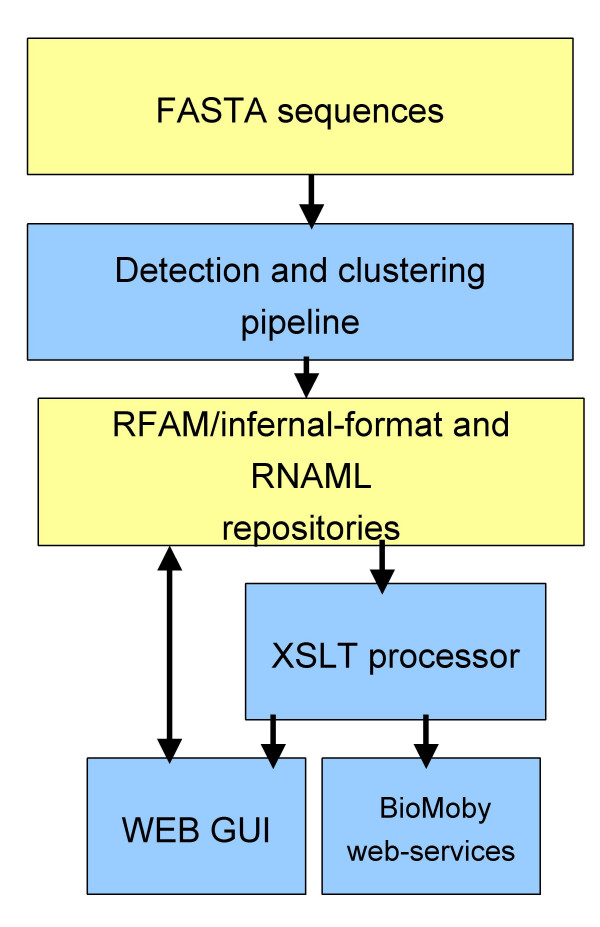
**Overview of the package architecture**. The diagram represents the general architecture of the software. Yellow boxes represent data and blue boxes processing. Internally, LeARN relies on RFAM and RNAML repositories for better data exchange with existing tools.

The general architecture of LeARN is shared by most annotation platforms (Figure 1). The first component is a perl pipeline which combines the analysis of genomic sequences in FASTA format with the integration of results (filtering, clustering, secondary structure prediction, etc.). Without further configuration, the current version relies on three different detection programs: rfam_scan.pl to scan the Rfam database; blastn to scan a database of large RNA molecules, and tRNAScan-SE for tRNAs. Moreover, it can easily be customized in order to add arbitrary detection software generating GFF2 output files. The data storage layer is implemented both as a repository of RNAML files and as a Rfam-formatted database. This redundancy guarantees interoperability with other software using the RNAML syntax as well as with infernal and related programs for searching and building families. The interface layer provides the user with interactive annotation sheets allowing the expert annotation of single RNA molecules or RNA families. The web server is implemented in perl-CGI. While data access for visualisation is implemented using an XSLT processor and XSL stylesheets, perl-object modules are used for repository modification. In addition, the package includes perl templates to provide access to the LeARN instances via BioMoby web-services.

### Set-up

The package (Additional file 1) provides command line programs for setting up and managing LeARN in the context of annotation projects. These programs (i) ensure the set-up of a new LeARN instance by generating site specific configuration files, and check the existence of mandatory binaries; (ii) manage user authentication required for annotation editing; (iii) allow new detection software to be registered in the LeARN pipeline and (iv) modify the release browsed by the web server.

## Results and discussion

### Detection and clustering pipeline

**Figure 2:**
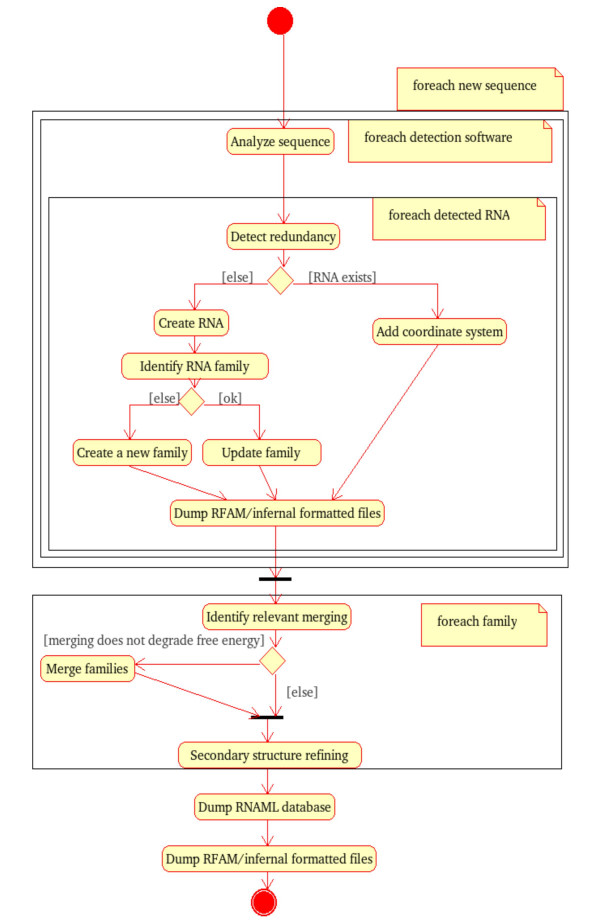
**Activity diagram of the detection and clustering pipeline**. The diagram shows the three main steps in the LeARN pipeline. It starts with the greedy ncRNA candidates clustering process, followed by the family refining step and the final dump of repositories.

The LeARN pipeline (Figure 2) integrates the results of detection programs. The integration is performed by a greedy algorithm which iteratively analyses ncRNA candidates provided by detection software. In the pipeline, each candidate is tested against pre-existing LeARN families (using rfam_scan.pl, blastn, cmsearch). When the molecule is detected as a member of an existing family, the family is updated, re-aligned (clustalw), folded (rnaalifold) and a new covariance model is built (cmbuild), otherwise a new singleton family is created. The greedy process stops when all detected candidates have been evaluated.

Because of its greediness, and depending on the order in which candidates are evaluated, family identification may be unsatisfactory. If the seed is far from the family consensus, the covariance model is not sensitive enough to capture the other members of the family. This lack of sensitivity may lead to a fragmentation of the families. Therefore, a post-process aimed at clustering subfamilies follows the analysis of candidates. This refinement process starts by a blastn search to identify families which are candidates for merging on the basis of sequence similarity. Then, pairs of sequence-related families are clustered, aligned and the free-energy of the secondary structure is computed using rnaalifold program. The clustering is validated only if the merged family does not degrade the free energy compared to isolated subfamilies.

#### Redundancy management

The algorithm manages two types of redundancies. The first redundancy is generated by the fact that different detection programs can detect the same type of ncRNAs (for example rfam_scan.pl and tRNA-ScanSE are both able to detect tRNA genes). LeARN handles this redundancy by prioritizing the detection software in the main configuration file. A second type of redundancy is caused by overlaps between sequences, and this is critical in the context of ongoing BAC-to-BAC sequencing projects. In order to manage this source of redundancy, LeARN can rely on an additional file describing pairwise overlaps to avoid artificial over-prediction of redundant ncRNA genes.

#### Incremental updates

The software allows for incremental updates of gene and family annotations which is an essential feature for ongoing sequencing projects. Incremental updating is made possible by using the RNAML files of the release *n-1 *before starting the greedy analysis of the release *n*.

#### Parallelization

In order to analyze one or several complete genomes, it is often useful to run detection computations in parallel. To accommodate the parallelization and the greedy algorithm previously described, the program offers the possibility to execute all detection programs beforehand. In this case, a command line is generated for each execution of a detection program. Each command line stores the result of its execution in a cache directory using an unambiguous filename based on the program name and version and the MD5 checksum of the analyzed sequence and can be directly executed in parallel. When the pipeline later requires the execution of a prediction program, it may directly use the cache result instead of running the prediction itself.

#### Customization

The pipeline can easily be customized to integrate arbitrary detection programs providing results in GFF2 format. In addition to the definition of site specific pipelines, this opens the possibility of using LeARN as a light-weighted visualisation interface for researchers willing to develop new detection software.

### Database and interoperability

LeARN relies on a repository of RNAML documents to store the annotations of molecules and ncRNA families. This technical choice is compatible with the limited amount of data generated to annotate ncRNA (less than 10 Mb of RNAML to describe the annotation of 130 Mb of legume genome sequences). Relying on RNAML documents (i) provides a native interoperability with the visualisation software which use this standard format; (ii) takes advantage of XSLT processors which allow both document transformations and efficient (via XPATH queries) searches in XML repositories, and (iii) provides users with a light-weight package that does not require any RDBMS skill.

### User interface

The Web interface of LeARN is structured by the different functionalities it offers: scanning the current database and annotating ncRNA molecules or families.

#### Scan/Browse

**Figure 3:**
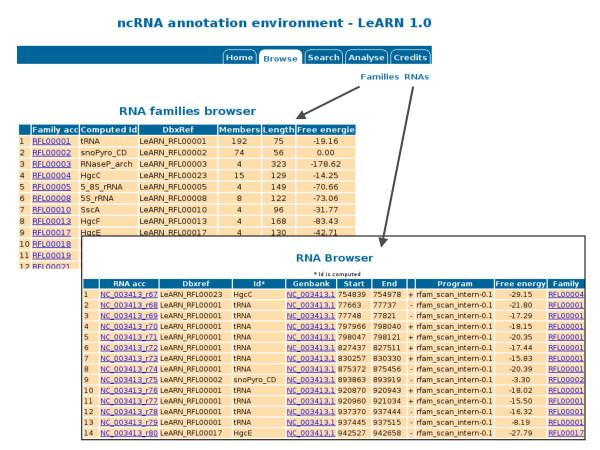
**RNA gene and RNA family browser**. The two main browsing pages allow access to either whole ncRNA families (left) or individual ncRNAs (bottom).

**Figure 4:**
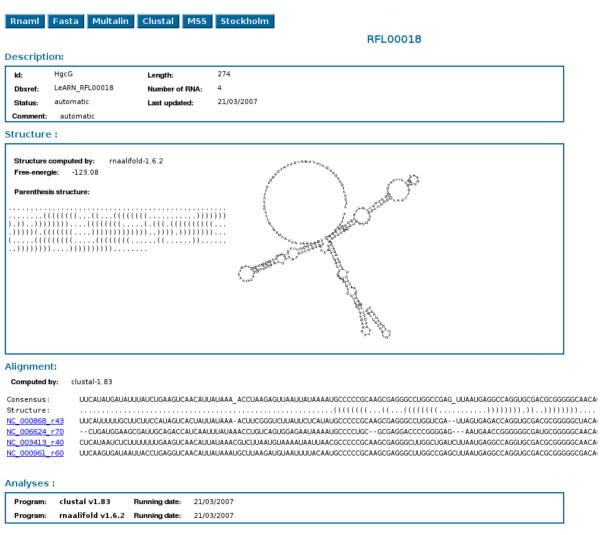
**Family entry view**. The sheet displays annotation, structure, alignment as well as links providing extracted information in standard formats (RNAML, multifasta, multalin, clustalw, MSS, Stockholm).

**Figure 5:**
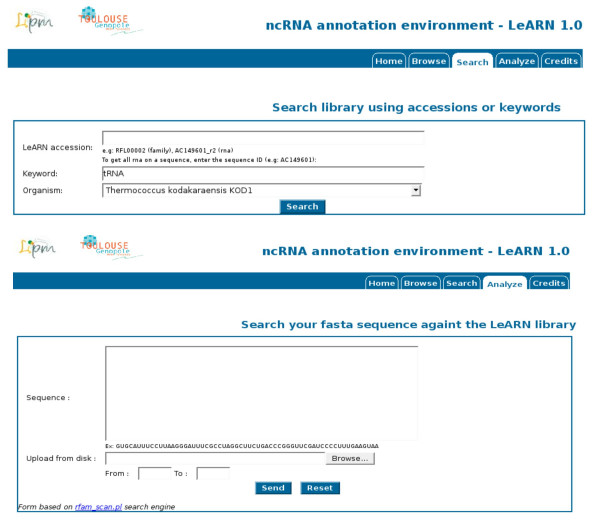
**Search forms**. LeARN provides forms to either query the RNAML repository using keywords or accession numbers (up, using the "Search" tab) or by using the sequence of a candidate ncRNA gene that should be annotated using the database (Analyze).

The "browse" tab allows lists of ncRNAs and ncRNA families to be displayed (Figure 3). By clicking on any ID, the user can access the structural and functional annotation of the molecules (Figure 4). In addition, the web interface provides different forms to query the database (Figure 5). The first mode allows ncRNA molecules or families to be retrieved that match a given accession, keyword or are from a selected species. The second form runs the rfam_scan.pl search engine on a given sequence using the LeARN database as the target.

#### Interactive annotation

In order to correct the unavoidable discrepancies and errors generated by an automatic process, the LeARN package provides the expert user with an annotation interface for editing both structural and functional annotations as well as merging and splitting families. This requires user authentication. After the first login, the user must create his/her own workspace, initially defined by a copy of the public database. At any time, the 'Status' page allows the user to select the database he/she wants to use: it can be either the browsable public database or its own editable private one. Editing rights are made visible by a change in the background colour. The system allows for the parallel annotation by several experts, but prevents the concurrent annotation of the same family by different experts.

#### ncRNA annotation

**Figure 6:**
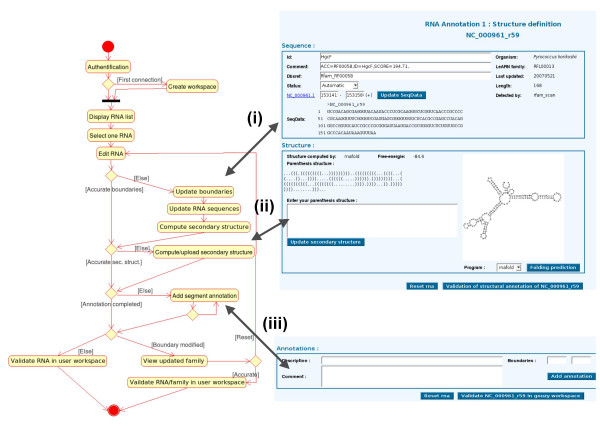
**The three steps of individual ncRNA edition**. The activity diagram (left) is related to the ncRNA edition page (right). The edition process goes through (i) boundary validation; (ii) secondary structure validation, and (iii) segment annotation.

In LeARN, the annotation of a ncRNA molecule is a three step process (Figure 6). The first step is the definition of the boundaries of the molecule and its description. Then, the annotator must provide the secondary structure of the ncRNA which can either be computed by on-line programs (mfold and rnafold) or pasted in a dedicated form when the results of on-line programs do not provide a satisfactory output. Optionally, the latest stage of the annotation is the annotation of the molecule itself (such as identification of the mature micro RNA on its precursor).

#### ncRNA family annotation

**Figure 7:**
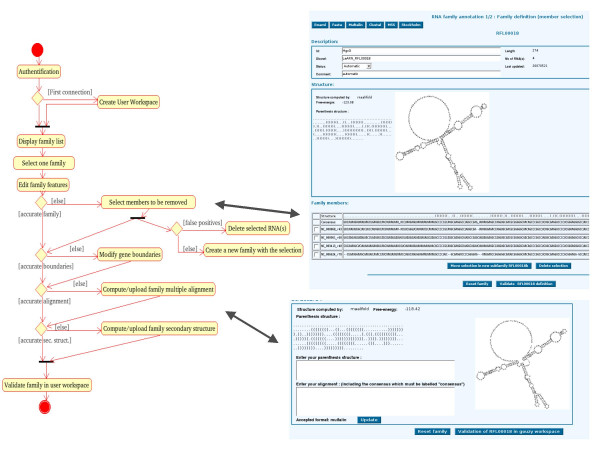
**ncRNA family edition**. The activity diagram (left) is related to the two ncRNA family edition pages (right). The first page (top right) captures the family members selection process and the second page (bottom right), captures the family annotation process, including both multiple alignment modification and secondary structure edition.

The annotation process for families is similar to the previous one (Figure 7). The first step of the family annotation is the selection of the members belonging to the family. This can be done either by merging RNA families or by removing false positive members of an existing family (any RNA belongs to a family which can be a singleton). The second step corresponds to the edition and validation of the boundaries of individual ncRNAs. Then, the multiple alignment of the family can be computed by on-line software (clustalw) or pasted in the appropriate form (if the previous alignment is unsatisfactory). Once the alignment has been validated, the annotator must validate the corresponding secondary structure which is automatically computed (rnaalifold) and editable.

### Case studies

We illustrate LeARN usage with two case studies on thermoccocales genomes *Pyrococcus abyssi *(Pa), *Pyrococcus furiosus *(Pf), *Pyrococcus horikoshii *(Ph) and *Thermococcus kodakarensis *(Tk). For these genomes, the RFAM genome browser gives 11 sRNA families in Pa, 12 sRNA families in Pf, 10 sRNA in families in Ph and 10 sRNA families in Tk for a total of 13 different families. With LeARN, iteratively built with RFAM covariance models, a classification in 16 families was obtained. From the original RFAM families, only the snoR9 family disappeared in this classification.

#### C/D box sRNA family annotation

We chose the C/D box sRNA family as the first case study. This family is mainly characterized by the presence of four motifs: C (RUGAUGA), D' (CUGA), C' (UGAUGA) and D boxes (CUGA). The region of 9 nucleotides downstream of D and/or D' boxes generally interacts with the target of the C/D sRNA mostly forming Watson-crick interactions. For a more accurate annotation, it is usual to further classify these ncRNA genes into subfamilies with a common target. In LeARN, four families were built automatically from the RFAM covariance model associated to the C/D box sRNA family (ID: snoPyro_CD). The largest snoPyro_CD family contained 74 candidates while the other ones contained only 1 candidate. From the Family browser, it was possible to select and merge all the four families (RFL0002, RFL0024, RFL0027, RFL0028) into only one (RFL0002) making it possible to analyze all candidates together and to classify them according to the conserved boxes and the sequence similarity at the target interaction site. This was done on the merged family using the Edit family function. Using the incrementally updated alignments provided by LeARN, the clustering of sequences into subfamilies based on their targets was straightforward. At the end of the process, 23 novel families were proposed. We renamed them according to the archaea sRNA database [32]. Three candidates remained in RFL0002. Remarkably, these three candidates correspond to the three families that contained only one candidate at the beginning of the annotation process. They were classified by the annotator as false positive candidates. Another interesting result of this annotation was the classification of sR9 in RFL0002. Indeed in RFAM, sR9, which contains both a C/D box and an H/ACA sRNA constitutes the snoR9 family and contains a candidate for each of the four organisms. The C/D box region of snoR9, corresponding to the sR9 C/D box sRNA, is also contained in the snoPyro_CD family of RFAM. This snoR9 family was lacking in LeARN at the beginning of the annotation process. It was manually created by the annotator and named sR9. In LeARN, all the organisms had a sR9 candidate, but only two of them contained both the C/D box and the H/ACA sRNA regions. The remaining ones lacked the H/ACA region. Thanks to the RNA edit function, it was possible to extend the 5' region of sRNA for which the H/ACA region was missing. Thus it was possible to verify and validate the presence of the H/ACA missing region before the C/D box sRNA region. Once the region had been inserted and validated, each new sRNA was updated in the family yielding a new alignment and structure for this family. The resulting structure contained the consensus hairpin structure of the H/ACA region for the four sRNA candidates. Overall, one should note that 23 families were found. Among these 23 families, one family (sR22) is not represented in the full alignment of RFAM. But these 23 families should be compared to the 60 families defined in the archaea snoRNA database. The missing 37 families of C/D box sRNA, representing probably more than 120 sRNA, are not detected by the RFAM tools and therefore do not appear in LeARN. From this we conclude that more sensitive tools are needed to find members of this family.

#### H/ACA sRNA family annotation

The second case study concerns H/ACA sRNA. In RFAM, HgcE, HgcF and HgcG have unknown function but were found to be H/ACA sRNA genes named respectively Pf3, Pf6 and Pf7 [33]. Thanks to the graphical representation of the secondary structure associated to the consensus of each family, it was immediately visible that the secondary structure of most of these sRNA was inaccurate. With LeARN, we could easily correct the alignment and structure. The structures of Pf7 (HgcG) and Pf6 (HgcF) were edited to contain three hairpins each, as described in [33]. The known structure of Pf3 (HgcE) includes two hairpins, as described in [33]. In RFAM and therefore also in LeARN, the sequence of members of the HgcE family contain only half of the first hairpin and the second hairpin. With the RNA edit function, it was simple to extend each sRNA to include the missing region. It was also necessary to edit the family in order to improve the automatically provided alignment. The result is now a more accurate annotation of sequences, structures and functions of HgcE, HgcF and HgcG sRNA.

To summarize, LeARN provides a way to complete the gap between existing databases and gene prediction tools. It offers the user a working environment for editing sequence and structure of individual sRNA, as well as RNA families, by using sRNA dedicated functionalities for automatic and manual annotation operations.

The first case study showed that one of the advantages of LeARN is to manage redundancy of candidates. The case of sRNA sR9 is a good example. One of the drawbacks of the automatic process of sRNA identification was the assignment of the sR9 C/D box sRNA to the general snoRNA family instead of the more accurate snoR9 family provided by the covariance model of RFAM. This is certainly a result of the proposed iterative approach which relies in part on the energy heuristic clustering. Despite this wrong assignment and the incomplete sequence for two members of this family, it was possible to group all the sR9 candidates and to extend those for which the H/ACA region was lacking by using the various functionalities of LeARN. In both case studies, it was particularly useful to be able to edit a sequence (extension of some regions) with regard to the literature and available knowledge of known H/ACA sRNA, candidates of the family, and availability of genomic sequences. The availability of an automatic or manual alignment of sRNA of the sequence and the structure, and the graphical representation of the consensus secondary structure considerably facilitated the improvement of the structural annotation.

All the annotations were done and saved in the private environment of the annotator which offers a very nice and useful working space for personal annotation. After the annotation, it appears now essential to submit these structural annotations to the administrator to replace less accurate ones. One can also imagine that these annotations could be submitted, in the context of a collaborative annotation process, to any RNA database administrator in order to share the annotations with the scientific community. For example, novel sequence and structural annotations of C/D box and H/ACA archaea sRNA could allow new, more accurate RFAM covariance models to be generated for future archaea genome annotations.

The demo section of the LeARN home page provides access both to raw results generated by the automatic process applied on the four thermoccocale genomes ("demo server"), and to the database after the edition of C/D box and H/ACA sRNA families ("annotation of C/D box and H/ACA families").

## Conclusion

The LeARN package can be used for ncRNA annotation projects for any set of sequences including complete genomes. It integrates tools and web interfaces covering the three layers of the ncRNA annotation process: a flexible detection and clustering pipeline, a RNAML database and a web interface to manage the expert annotation. The software has been designed to manage the complete ncRNA annotation process in the frame of whole genome annotation projects and to fill the gap between existing detection software and public ncRNA repositories. Moreover, LeARN is also an extendible and light-weight package, which can be used as an annotation interface either by life scientists wanting to annotate a single ncRNA family or by bioinformaticists who need a simple interface to visually evaluate their results. It can also be used to build training datasets or for any other activity involving ncRNA annotation.

## Availability and requirements

**Project name**: LeARN

**Project home page**: 

**Operating system**: UNIX

**Programming language**: Perl-OO, XSLT

**Other requirements**: Infernal [9], Vienna package [34], Mfold [35], Rfam [8], tRNAScan-SE [18], ClustalW [36], ncbi-blast [37]. The AdminGuide reports the full list of dependencies.

**License**: Free Software license CECILL2 

**Any restrictions to use by non-academics**: None

## List of abbreviations

XML: eXtensible Markup Language

XSLT: eXtensible Stylesheet Language Transformations

## Authors' contributions

CN designed, implemented and tested the software; JG led the design. JG, CG and TS tested the programs and contributed to the preparation of the manuscript. All authors have read and approved the final manuscript.

## Supplementary Material

Additional file 1 **LeARN 1.0.1 tarball**. Tarball with LeARN source code. For installation instructions, see . Click here for file
