# Supplementary material for: LeARN: a platform for detecting, clustering and annotating non-coding RNAs
Source: BMC Bioinformatics. 2008 Jan 14;9:21. doi: 10.1186/1471-2105-9-21 (PMC2241582; doi:10.1186/1471-2105-9-21)
Supplement: Additional file 1 — LeARN 1.0.1 tarball. Tarball with LeARN source code. For installation instructions, see . [file 1471-2105-9-21-S1.GZ › LeARN/doc/AdminGuide-1.0.1.pdf]

LeARN 1.0.1

November 2007

Céline Noirot, Christine Gaspin, Thomas Schiex, Jérôme Gouzy

learn@toulouse.inra.fr

Admin Guide

Table of Contents

LeARN 1.0.1....1
Download....2
Install software prerequisites....2
Edit the configuration file....5
Configure the instance....5
Check Perl module installation....5
Create configuration files....5
Add a new software [optional]....6
Create user accounts....6
Initialize the web server....7
Create the first release....7
[OPTIONAL] Pre-computation....7
Run the clustering algorithm....7
Release update....8
Select/Set the default web release....8
QuickStart....9

Download

Create a LeARN directory on your hard drive and initialize the LEARN_DIR environment variable to this directory. Then download the most recent tarball.

```
% wget http://symbiose.toulouse.inra.fr/LeARN/download/LeARN-1.0.1.tar.gz
% gzip -cd LeARN-1.0.1.tar.gz | tar xvf -

% cd LeARN

% setenv LEARN_DIR /www/LeARN (csh/tcsh)
or
% export LEARN_DIR=/www/LeARN (sh/bash)
```

Install software prerequisites

LeARN uses external programs which must be either installed or linked to $LEARN_DIR/bin/ext/bin.

- xsltproc: http://xmlsoft.org/XSLT/xsltproc2.html
- ImageMagick: http://www.imagemagick.org/
- wget: http://www.gnu.org/software/wget/
- bioperl >=1.4
- perl >=5.6 and modules http://www.cpan.org/
  - XML::Simple
  - LWP
  - Class::XML
  - Class::Accessor
  - Class::Data::Inheritable
  - XML::XPath
  - XML::Twig
  - XML::TreeBuilder
  - Convert::UU
  - HTML::Entities
- clustalw: http://www.cf.ac.uk/biosi/research/biosoft/Downloads/clustalw.html

– mfold: http://www.bioinfo.rpi.edu/~zukerm/export/

– ncbi-blast ftp://ftp.ncbi.nlm.nih.gov/blast/executables/release

– multalin: ftp://ftp.toulouse.inra.fr/pub/multalin
– tRNAScanSE: ftp://ftp.genetics.wustl.edu/pub/eddy/software/tRNAscan-SE.tar.Z
– infernal: ftp://selab.janelia.org/pub/software/infernal/infernal.tar.gz
– Vienna package: http://www.tbi.univie.ac.at/~ivo/RNA/
– rfam_scan.pl: http://www.sanger.ac.uk/Software/Rfam/help/scripts/search/rfam_scan.pl
– rfam: ftp://ftp.sanger.ac.uk/pub/databases/Rfam/Rfam.full.gz

To permit tracability over years of the annotation process, LeARN links all analyses to its program (name+version). But to ensure over years the consistency of this information, the LeARN admin must have a full control of the program versions, it means that at any time he/she must be certain of which release of program he/she is running. To do so we suggest to install (and to keep) all releases of the programs inside the $LeARN_DIR/bin/ext directory.

The main programs used by LeARN are infernal, tRNAScan-SE, the vienna package, mfold and ncbi-blast. Below we present how we install the main software inside the LeARN directory (tested with linux debian and ubuntu).

* Infernal

```
% cd $LEARN_DIR/bin/ext
% wget ftp://selab.janelia.org/pub/software/infernal/infernal-0.72.tar.gz
% gzip -cd  infernal-0.72.tar.gz | tar xvf -
% cd $LEARN_DIR/bin/ext/infernal-0.72
% more LICENSE
%./configure --prefix $LEARN_DIR/bin/ext
% make
with infernal-0.7/gcc version 4.0.3 (Debian 4.0.3-1) erreur de compilation
gcc -I. -g -O2  -c easel.c
easel.c:13: error: static declaration of 'esl_error_handler' follows non-static declaration
./easel.h:140: error: previous declaration of 'esl_error_handler' was here
make: *** [easel.o] Error 1

=> edit easel.h to comment the line 140
/*extern esl_error_handler_f esl_error_handler;*/
% make
% make install
% cp squid/sreformat $LEARN_DIR/bin/ext/bin
```

* tRNAscan-SE

```
% cd $LEARN_DIR/bin/ext
% wget  ftp://ftp.genetics.wustl.edu/pub/eddy/software/tRNAscan-SE.tar.Z
% zcat tRNAscan-SE.tar.Z | tar xvf -
```

```
% cd $LEARN_DIR/bin/ext/tRNAscan-SE-1.23
% more GNULICENSE
% setenv HOMEBK $HOME ; export HOMEBK=$HOME
% setenv HOME $LEARN_DIR/bin/ext ; export HOME=$LEARN_DIR/bin/ext
% make
% make install
% setenv HOME $HOMEBK ; export HOME=$HOMEBK
```

* MFOLD / MFOLD_UTIL (require gcc, g++ and g77)

```
% cd $LEARN_DIR/bin/ext
% wget http://www.bioinfo.rpi.edu/~zukerm/export/mfold-3.2.tar.gz
% gzip -cd mfold-3.2.tar.gz | tar xvf -
% cd $LEARN_DIR/bin/ext/mfold-3.2
% more Academic_License.txt
% setenv BINDIR $LEARN_DIR/bin/ext/bin; export BINDIR=$LEARN_DIR/bin/ext/bin
% setenv DATDIR $LEARN_DIR/bin/ext/dat; export DATDIR=$LEARN_DIR/bin/ext/dat
% mkdir -p $BINDIR $DATDIR
% make -e install

% cd $LEARN_DIR/bin/ext
% wget http://www.bioinfo.rpi.edu/~zukerm/export/mfold_util-3.3.tar.gz
% gzip -cd mfold_util-3.3.tar.gz | tar xvf -
% cd mfold_util-3.3
% more LICENSE
% ./configure --prefix $LEARN_DIR/bin/ext
% make install
```

* Vienna Package

```
% cd $LEARN_DIR/bin/ext
% wget http://www.tbi.univie.ac.at/~ivo/RNA/ViennaRNA-1.6.2.tar.gz
% gzip -cd ViennaRNA-1.6.2.tar.gz | tar xvf -
% cd $LEARN_DIR/bin/ext/ViennaRNA-1.6.2
% more COPYING
% ./configure --prefix $LEARN_DIR/bin/ext --without-kinfold --without-forester --
without-perl
% make install
```

* NCBI-BLAST

```
% cd $LEARN_DIR/bin/ext
% wget ftp://ftp.ncbi.nlm.nih.gov/blast/executables/release/2.2.15/blast-2.2.15-
ia32-linux.tar.gz
% gzip -cd blast-2.2.15-ia32-linux.tar.gz | tar xvf -
% ln -s blast-2.2.15 ncbi-blast
% cp blast-2.2.15/bin/blastall blast-2.2.15/bin/formatdb $LEARN_DIR/bin/ext/bin
```

* CLUSTALW

```
% cd $LEARN_DIR/bin/ext
% wget ftp://ftp.ebi.ac.uk/pub/software/unix/clustalw/clustalw1.83.UNIX.tar.gz
% gzip -cd clustalw1.83.UNIX.tar.gz | tar xvf -
% cd  clustalw1.83
% more README
% make
% cp  clustalw $LEARN_DIR/bin/ext/bin
```

* Multalin

```
% cd $LEARN_DIR/bin/ext
% wget ftp://ftp.toulouse.inra.fr/pub/multalin/multalin.5.4.1/multalin.LINUX.Z
% mkdir multalin
% cd multalin
% zcat ../multalin.LINUX.Z | tar xvf -
% cp ma $LEARN_DIR/bin/ext/bin
```

*** JgoughPhylo.pm**

```
% cd $LEARN_DIR/lib
% wget http://supfam.mrc-lmb.cam.ac.uk/treedraw/JgoughPhylo.pm
```

Edit the configuration file

Edit the file `$LEARN_DIR/cfg/Template_Learn.cfg` to search all keys containing the strings "_cmd" and "_version". Then you must modify the values of the command line (_cmd) and the values of the program versions (_version) according to your local configuration. At that time you can decide to use either the binaries installed into your LeARN directory (refered as MASK_LEARN_DIR) or elsewhere on your hard drive (specifying a complete path). In both cases you can decide to specify either a directory including the program version as MASK_LEARN_DIR/bin/ext/infernal-0.7/bin/cmsearch (better for tracability in our opinion) or without the version MASK_LEARN_DIR/bin/ext/bin/cmsearch or /usr/local/bioinfo/bin/cmsearch)

Configure the instance

Check Perl module installation

Run the following command line to check if all mandatory perl modules are installed.

```
% $LEARN_DIR/bin/EvalPerlModule.pl
```

The program fails if a module is missing. Install all mandatory modules until it prints « OK ».

Create configuration files

```
$LEARN_DIR/bin/LeARN_Install.pl --learn_dir $LEARN_DIR --learn_url
http://localhost/LeARN
```

```
Create : /www/LeARN/cfg/Learn.cfg
Create : /www/LeARN/cfg/LearnCgi.cfg
Create : /www/LeARN/lib/Var.pm
Create : /www/LeARN/web/xml/learn.xml
Create : /www/LeARN/cfg/RNAPlotEval.cfg
Create : /www/LeARN/cfg/RNALocalFold.cfg
Create : /www/LeARN/cfg/ForCgi.cfg
```

This installation program tests that programs specified in the configuration file exist on your system and fails if not. When it fails, you must correct the error and run again the program until it completes without any error.

Add a new software [optional]

LeARN allows the integration of any detection software providing results in the standard GFF format release 2.

```
AC146854.19     Rfam     domain   84517    84590    .       +       .       Att "ACC=RF00005;ID=tRNA;SCORE=62.95;"
AC146854.19     Rfam     domain   48268    48370    .       +       .       Att "ACC=RF00451;ID=mir-395;SCORE=41.08;"
AC146854.19     trnascan          tRNA     84517    84590   .       +       .       Att "Val;AAC;SCORE=76.95"
```

The other constraint is that your program must have the following usage:

```
my_wonderful_program « any parameters » --input file --output file.out
```

When your program does not have a valid output format and/or a valid usage the easier way to integrate it into LeARN is to embbed the program in a perl/python script which match the requirements.

When the requirements are solved, you must register the additional software following the example below:

```
$LEARN_DIR/bin/LeARN_AddSoftware.pl          --name=snoscan          --cmd=$LEARN_DIR/bin/get_SnoScan.pl  --param  "--cfg  $LEARN_DIR/cfg/get_snoscan.cfg" --version=0.01
```

The script will ask for a priority value for the program. This priority is critical to manage redundancy when several detection programs predict the same ncRNA.

Finally, you must run again `$LEARN_DIR/bin/LeARN_Install.pl` in order to take into account the new program.

Create user accounts

```
$LEARN_DIR/bin/LeARN_AddUser.pl
        --help            : this message
        --login string    : login of the user
        --email string    : email of the user
        --privilege char  : number [0|1]
```

Example:

```
$LEARN_DIR/bin/LeARN_AddUser.pl --login alien --email alien@mars.org
```

Warning: the installation program creates two demo user accounts

`login: demo1 password: demo1 (with guest privilege)`

`login: demo2 password: demo2 (with annotator privilege)`

If you do not want to use these accounts, you must delete the corresponding lines in `$LEARN_DIR/data/auth.priv`

Initialize the web server

Add the directives in your web server configuration file. For apache, add the

lines below by replacing /www/LeARN by the content of your $LEARN_DIR

```
<Directory "/www/LeARN/">
    Order deny,allow
    Deny from all
</Directory>

<Directory "/www/LeARN/cgi-bin/">
    Options +ExecCGI
    Order deny,allow
    Allow from all
</Directory>

<Directory "/www/LeARN/web/">
    Options +FollowSymLinks
    Order deny,allow
    Allow from all
</Directory>

<Directory "/www/LeARN/data/">
    Options +FollowSymLinks
    Order deny,allow
    Allow from all
</Directory>

ScriptAlias /LeARN/cgi-bin/ /www/LeARN/cgi-bin/
AddHandler cgi-script .cgi
```

Remark: the more efficient is to ask your system manager to check/adapt your configuration.

Create the first release

You can only run the analyze and store the result in a repository or launch the complete analyze and build the LeARN database.

[OPTIONAL] Pre-computation

On large datasets it is required to parallelize the computation of detection software. This program build a file which contain the list of “atomic” command lines. These commands can be easily run on a cluster.

```
Usage:./bin/LeARN_BuildScanCli.pl
  --help                          this message
  --cfg  filename                 full path of the configuration file
  --path_old_release dirname      path of old release
  --path_new_release dirname      path of new release (to create)
  --input_fasta filename          multifasta input file of one species
  --species quoted_string         name of the organism , eg: 'Casimir vulgaris'
  --log filename                  log file
```

Run the clustering algorithm

This can reuse the precomputed analyses if you define the same repository as for the pre-computation step.

```
$LEARN_DIR/bin/LeARN.pl
  --help  :               this message
[Mandatory]
  --path_old_release dirname : path of the previous release
  --path_new_release dirname : path of the new release (to create)
  --input_fasta      filename: multifasta input file belonging to one species
  --species   'quoted string': name of organism , eg: 'Medicago truncatula'
  --cfg              filename: configuration file
[Optional]
  --upgraded_sequences       : when some versions of BAC are upgraded specify the
file containing the list correspondances: old_bac_accession new_bac_accession
  --overlaps filename        : file containing overlap data : [seq1 start1 end1
strand1 seq2 start2 end2 strand2]
  --log  filename            : log file
```

Example:

```
% export RFAM_DIR=$LEARN_DIR/data/release_template/db/Rfam/ # or any other Rfam
directory
% $LEARN_DIR/bin/LeARN.pl \
                       --path_new_release $LEARN_DIR/data/v1 \
                       --species "Casimir vulgaris" \
                       --input_fasta Cv.multifasta
```

Release update

For updating the database, either with a dataset of the same species or using sequences of another species you must run the program by providing both a directory for the new release and the directory of the previous release.

```
% export RFAM_DIR=$LEARN_DIR/data/release_template/db/Rfam/ # or any other Rfam
directory
% $LEARN_DIR/bin/LeARN.pl  \
                      --path_old_release $LEARN_DIR/data/v1  \
                      --path_new_release $LEARN_DIR/data/v2  \
                      --species "Casimir singularis" \
                      --input_fasta Cs.multifasta
```

Select/Set the default web release

```
$LEARN_DIR/bin/LeARN_SetWebRelease.pl

        --help                  : this message
        --root dirname          : default is $LEARN_DIR
        --release_dir dirname   : relative to $LEARN_DIR dir eg: data/rel_template
        --new                   : initialize a web site for testing a new release
```

Example:

```
$LEARN_DIR/bin/LeARN_SetWebRelease.pl --release_dir data/v1
```

The parameter –-new build a temporary web site release but do not modify the default web release. This feature is useful for testing a new release without modifying the default.

Example:

```
$LEARN_DIR/bin/LeARN_SetWebRelease.pl --release_dir data/v2 --new
```

QuickStart

Considering that you have successfully installed a "LeARN" instance, here are the commands that you can run in order to create your own demo web server. The "LeARN" demo server corresponding to the automatic analysis of 4 archea genomes: *Pyrococcus abyssi, Pyrococcus horikoshi, Thermococcus kodakaraensis KOD1, Pyrococcus furiosus.*

1) Fetch the data and copy the fasta files in your $LEARN_DIR directory as defined during the set-up (e.g /www/LeARN/)

```
cd $LEARN_DIR/data

wget ftp://ftp.ncbi.nlm.nih.gov/genomes/Bacteria/Pyrococcus_abyssi/NC_001773.fna
wget ftp://ftp.ncbi.nlm.nih.gov/genomes/Bacteria/Pyrococcus_abyssi/NC_000868.fna
cat NC_001773.fna NC_000868.fna > Pyrococcus_abyssi.fna

wget ftp://ftp.ncbi.nlm.nih.gov/genomes/Bacteria/Pyrococcus_horikoshii/NC_000961.fna
mv NC_000961.fna Pyrococcus_horikoshii.fna

wget ftp://ftp.ncbi.nlm.nih.gov/genomes/Bacteria/Pyrococcus_furiosus/NC_003413.fna
mv NC_003413.fna Pyrococcus_furiosus.fna

wget ftp://ftp.ncbi.nlm.nih.gov/genomes/Bacteria/Thermococcus_kodakaraensis_KOD1/NC_006624.fna
mv NC_006624.fna Thermococcus_kodakaraensis_KOD1.fna
```

2) Run the pipeline on the 4 genomes (~ 3/4hours depending your hardware)

```
cd $LEARN_DIR

$LEARN_DIR/bin/LeARN.pl --path_new_release $LEARN_DIR/data/relTherm1 –input \
   data/Pyrococcus_horikoshii.fna --species "Pyrococcus horikoshii" --log log/relTherm.1.log

$LEARN_DIR/bin/LeARN.pl --path_old_release $LEARN_DIR/data/relTherm1 –path_new_release \
   $LEARN_DIR/data/relTherm2 --input data/Pyrococcus_abyssi.fna --species "Pyrococcus abyssi" –log \
   log/relTherm.2.log

$LEARN_DIR/bin/LeARN.pl --path_old_release $LEARN_DIR/data/relTherm2 –path_new_release  \
   $LEARN_DIR/data/relTherm3 --input data/Pyrococcus_furiosus.fna --species "Pyrococcus furiosus"  \
   --log  log/relTherm.3.log

$LEARN_DIR/bin/LeARN.pl --path_old_release $LEARN_DIR/data/relTherm3 –path_new_release \
   $LEARN_DIR/data/relTherm4 --input data/Thermococcus_kodakaraensis_KOD1.fna --species \
   "Thermococcus kodakaraensis KOD1"  --log log/relTherm.4.log
```

3) Add a new user with editing privilege

```
$LEARN_DIR/bin/LeARN_AddUser.pl --login therm --email name@domain.org --privilege 1 --passwd
motdepasse
```

4) Select the latest release as the default web release

```
$LEARN_DIR/bin/LeARN_SetWebRelease.pl --root $LEARN_DIR --release_dir data/relTherm4
```

5) Access the web server (the url depends on your local installation)

At that stage you should be able to browse and query the database

6) Edit the database (in a private workspace)

* first click on Home/Connexion to enter the login (therm) and the password (motdepasse)

* then go to Home/Status to load a copy of the database in you private workspace

* after that step, you can select either the public database for browsing or select the private one to edit ncRNA and/or families
