# Supplementary figures and images for: LeARN: a platform for detecting, clustering and annotating non-coding RNAs
Source: BMC Bioinformatics. 2008 Jan 14;9:21. doi: 10.1186/1471-2105-9-21 (PMC2241582; doi:10.1186/1471-2105-9-21)

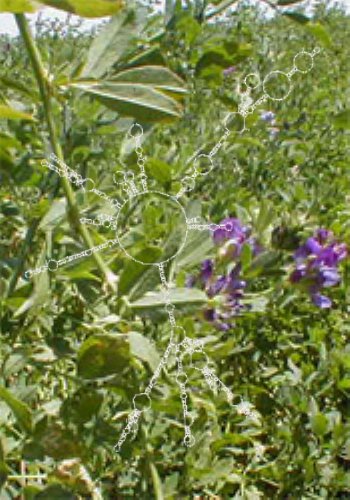

Supplement: Additional file 1 — LeARN 1.0.1 tarball. Tarball with LeARN source code. For installation instructions, see . [file 1471-2105-9-21-S1.GZ › LeARN/web/img/LeARNHomePageV3.jpg]
